# Corticosteroid injection plus exercise versus exercise, beyond advice and a heel cup for patients with plantar fasciopathy: protocol for a randomised clinical superiority trial (the FIX-Heel trial)

**DOI:** 10.1186/s13063-019-3977-0

**Published:** 2020-01-02

**Authors:** Henrik Riel, Bill Vicenzino, Jens Lykkegaard Olesen, Martin Bach Jensen, Lars Holger Ehlers, Michael Skovdal Rathleff

**Affiliations:** 1 Center for General Practice at Aalborg University, Fyrkildevej 7, 9220 Aalborg East, Denmark; 2 The University of Queensland, School of Health and Rehabilitation Sciences: Physiotherapy: Sports Injury Rehabilitation and Prevention for Health, St. Lucia, QLD 4072 Australia; 3 Danish Center for Healthcare Improvements (DCHI), Department of Business and Management, The Faculty of Social Sciences, Aalborg University, Fibigerstræde 11, 9220 Aalborg East, Denmark; 4 Center for Sensory-Motor Interaction (SMI), Department of Health Science and Technology, Faculty of Medicine, Aalborg University, Fredrik Bajers Vej 7D, 9220 Aalborg East, Denmark; 5 Department of occupational therapy and physiotherapy, Aalborg University Hospital, Hobrovej 18-22, 9100 Aalborg, Denmark

**Keywords:** Plantar fasciopathy, Heavy-slow resistance training, Corticosteroid injection, Foot orthoses, Patient advice

## Abstract

**Background:**

Plantar fasciopathy has a lifetime prevalence of 10%. Patients experience sharp pain under the heel, often for several months or years. Multiple treatments are available, but no single treatment appears superior to the others. A corticosteroid injection offers short-term pain relief but is no better than placebo in the longer term (> 8 weeks). Heavy-slow resistance training has shown potentially positive effects on long-term outcomes (> 3 months), and combining exercises with an injection may prove to be superior to exercises alone. However, the effect of heavy-slow resistance training compared with a simpler approach of patient advice (e.g., load management) and insoles is currently unknown. This trial compares the efficacy of patient advice with patient advice plus heavy-slow resistance training and with patient advice plus heavy-slow resistance training plus a corticosteroid injection in improving the Foot Health Status Questionnaire pain score after 12 weeks in patients with plantar fasciopathy.

**Methods:**

In this randomised superiority trial, we will recruit 180 patients with ultrasound-confirmed plantar fasciopathy and randomly allocate them to one of three groups: (1) patient advice and an insole (*n* = 60); (2) patient advice, an insole, and self-dosed heavy-slow resistance training consisting of heel raises (*n* = 60); or (3) patient advice, an insole, heavy-slow resistance training, and an ultrasound-guided corticosteroid injection (*n* = 60). All participants will be followed for 1 year, with the 12-week follow-up considered the primary endpoint. The primary outcome is the Foot Health Status questionnaire pain domain score. Secondary outcomes include the remaining three domains of the Foot Health Status Questionnaire, a 7-point Global Rating of Change, the Pain Self-Efficacy Questionnaire, physical activity level, health-related quality of life measured by the EQ-5D-5L, and Patient Acceptable Symptom State, which is the point at which participants feel no further need for treatment. Additionally, a health economic evaluation of the treatments will be carried out.

**Discussion:**

This trial will test if adding heavy-slow resistance training to fundamental patient advice and an insole improves outcomes and if a corticosteroid injection adds even further to that effect in patients with plantar fasciopathy.

**Trial registration:**

ClinicalTrials.gov, NCT03804008. Prospectively registered on January 15, 2019.

## Background

One in ten persons will develop plantar fasciopathy (PF) [1, 2]. The condition accounts for 8% of all running-related injuries but is also common in the general population [3–5]. A high running volume is associated with a higher risk of developing PF, and despite a lack of prospective studies to identify risk factors among non-runners, a high body mass index is thought to be a risk factor [6]. Pain is often worse during the first steps after getting out of bed or during the first steps after periods of non-weight-bearing [7].

PF was formerly known as ‘plantar fasciitis’ or ‘heel spur syndrome’ and has historically been considered a self-limiting condition in which 80% are expected to achieve symptom-free status within 12 months. This view of a self-limiting condition has been challenged by research [1, 8–11]. Approximately half of patients referred to a specialised secondary care clinic still experienced pain 10 years after treatment [9], and 40% of patients in a randomised controlled trial still had symptoms 2 years after plantar fascia-specific stretching and wearing insoles [8]. Patients with PF show greater levels of depression, stress, anxiety and kinesiophobia and experience limitations in both mobility and health-related quality of life compared with pain-free individuals [12–14]. Moreover, PF may be associated with several days of sick leave. Davis et al. found that 6 of 105 patients had taken as much as 3 months off work [15], and we recently found that 23% of patients in the workforce had taken a median of 30 days off work due to their heel pain in one study [16] and 20% had taken between 5 and 548 days off work in another [17]. Thus, the consequences for both patients and society are marked.

A systematic review and network meta-analysis compared several commonly used treatment options for PF [18]. Overall, they concluded that none of the investigated treatments were superior to the others, but different treatments may have different temporal profiles. Some are efficacious in the short term (< 4 weeks), such as an injection with corticosteroid, whereas others (e.g., exercises or orthoses are more efficacious in the longer term [> 12 weeks]) [18–22]. Heavy-slow resistance training (HSR) was not included in the review, but it is generally known for a long-term efficacy in the rehabilitation of both upper- and lower-limb tendinopathies [23–25]. In PF, HSR has been found to be superior to plantar fascia-specific stretching, but only 6% achieve an acceptable symptom state within 12 weeks [16, 26]. This emphasises the need for additional improvements to current care of these patients.

Combining HSR with a corticosteroid injection may provide both short- and long-term pain relief for individuals with PF. We recently conducted a feasibility study of the combination of these two treatments, which supports running an efficacy trial. Patients’ rating of acceptability, time to commencement of exercise after the injection, compliance with exercise, recruitment rate, and changes in foot-related health and function over time supported this current proposed trial [27]. In theory, combined treatments could supplement each other and give both an immediate and long-term pain reduction.

Repeated corticosteroid injections and a combination of stretching and foot-strengthening exercises have been investigated before, but the combination of HSR and a single corticosteroid injection is yet to be studied [28]. Previous studies in other tendinopathies only compared the combined treatments with one of the treatments and reported no differences [24, 29]. The limitation of these studies is that it remains unknown if any of the treatments have had some effect or no effect overall on the condition, because the trials did not include a control arm [24, 29]. We propose that a trial of combined interventions versus one of the interventions requires a control arm. A minimal intervention (control arm) consisting of patient advice, which reflects current general practitioner (GP) practice (unpublished data), will enable a meaningful comparison with more time-consuming and expensive treatments, such as exercises and injections. If we do not fill this knowledge gap, we might use costly and time-consuming treatments without knowing the effect compared with simpler treatment. To our knowledge, there is no literature available on the cost-effectiveness of alternative treatments of PF [30]. Despite treatment costs for PF possibly seeming relatively small compared with other more expensive health technologies, the productivity costs to society may be substantial in PF due to patients’ sick leave.

The present trial is the product of a series of preceding studies conducted by our group in this patient population in general practice. Interviews with GPs about the management of PF showed a high heterogeneity wherein some would give a steroid injection at first consultation, some would prescribe exercises, and some would refer to a physiotherapist (unpublished data). However, commonalities were to give patients advice on what they could do to self-manage and an uncertainty about the effectiveness of the many treatments available for PF. To investigate if we could improve outcomes associated with HSR, we compared a traditional pre-determined HSR programme with a self-dosed programme to target self-efficacy and increase the exercise dose received. We found that both programmes were associated with similar improvements [16]. Following that randomised trial, we investigated the feasibility of combining HSR with an ultrasound-guided corticosteroid injection in the feasibility study described above as a final step before initiating the present trial [27].

### Objectives

The purpose of this trial is to investigate the efficacy of fundamental patient advice and a heel cup (PA) versus fundamental patient advice and a heel cup plus heavy-slow resistance training (PAX) versus a combination of fundamental patient advice and a heel cup plus heavy-slow resistance training and an ultrasound-guided corticosteroid injection (PAXI) in improving the Foot Health Status Questionnaire pain domain score after 12 weeks in individuals with PF.

#### Hypotheses

Hypothesis 1: The group receiving PAXI will be superior to the group receiving PAX.

Hypothesis 2: The group receiving PAXI will be superior to the group receiving PA only.

Hypothesis 3: The group receiving PAX will be superior to the group receiving PA only.

## Methods

### Design and setting

The FIX-Heel Trial is designed as a randomised superiority trial with a three-group parallel design. Reporting of the protocol follows the Standard Protocol Items: Recommendations for Interventional Trials (SPIRIT) statement (checklist uploaded as Additional file 1), TiDieR (Template for Intervention Description and Replication checklist and guide) and the Consensus on Exercise Reporting Template for intervention description [31–33]. The preparation of the trial, including publishing this trial protocol, was done in accordance with the PREPARE trial guide [34]. Before the inclusion of the first participant, the trial was registered with ClinicalTrials.gov (NCT03804008). Patients will be recruited from general practice and via social media (see below). However, for pragmatic reasons, the information and training instructions will be given at the physiotherapy department at Aalborg University Hospital, and injections will be given at a private rheumatology clinic (ReumaNord) situated in Aalborg, Denmark. Participants will attend the baseline and 12-week follow-up appointments at the hospital, whereas a link to the questionnaires used will be sent via REDCap (Vanderbilt University, Nashville, TN, USA) to participants’ e-mail addresses for the 4-week, 26-week and 52-week follow-ups.

### Roles and responsibilities

The project manager is a physiotherapist with 8 years of experience in treating patients with musculoskeletal conditions. He will be responsible for recruitment and analysing data whilst blinded to group allocation. Two physiotherapists working at the Aalborg University Hospital with 5 and 6 years of experience, respectively, were recruited and will be responsible for eligibility screening, delivering interventions and being in contact with participants throughout the trial. Before the inclusion of the first participant, the physiotherapists had received 10 and 19 h of training, respectively, in the procedures of the trial by the project manager. This includes putting four pilot study participants through all aspects of the trial processes. The ultrasound-guided injection will be performed by a rheumatologist who has more than 15 years of experience with performing ultrasound-guided injections.

### The adaptive recruitment strategy

Participants will primarily be recruited from general practice, but to ensure that the trial timeline is maintained, we will include participants through social media (Facebook). Initially, we aim to recruit from ten general practices in the North Denmark Region. We will reach out to the practices using an open invitation in which we ask for practices interested in being part of trial recruitment through the Facebook page of the Center for General Practice at Aalborg University and through our network of GPs in the North Denmark Region. If this is not sufficient, the project manager will contact general practices directly. The GPs are offered a 30-min presentation about PF and the trial at their own practice. If recruitment from general practice is inadequate, we may employ one of the practices’ secretaries, who examines the GPs’ daily plans for potential trial participants. The secretary will then remind the GP of informing the patient about the trial if the patient is potentially eligible for inclusion.

General practices will receive an honorarium per patient they refer to the project manager. It is a standard honorarium stipulated by the Danish Committee of Multipractice Studies in General Practice (approximately €18). Before they agree to participate in recruitment, they will be informed that they are expected to refer a minimum of two patients per month. On the basis of the number of referrals we receive, we will categorise general practices into three symbolic zones: green, yellow and red. Practices in the green zone will have referred a minimum of two patients during the past month; practices in the yellow zone will only have referred one patient during the past month; and practices categorised in the red zone will have failed to refer any patients during the past month or they will have been in the yellow zone for two consecutive months. Practices in the red zone will be contacted by the project manager to discuss whether other strategies are needed to increase referrals or if the reason for the lack of referrals is a lack of potentially eligible participants presenting at the practice. If half of the clinics are categorised in the red zone or if less than half of the referred patients are not eligible during a 2-month period, we will recruit additional general practices.

Recruitment through Facebook will be used if fewer than ten participants have been included during any given month following referrals from general practices. We will post information about the trial on a Facebook page called ‘Treatment of pains under the foot’ and use the sponsoring function. We target the sponsoring towards both males and females aged 18 years or older who are living within an 80-km radius of Aalborg, Denmark. Each sponsorship will last for 1 week. If this is not sufficient to achieve at least ten included participants per month, including those referred from general practices, we will increase the amount of money per sponsorship first and increase the radius second. Both strategies will increase the reach of the sponsorship. We are able to calculate the cost of each included participant recruited via Facebook by dividing the cost of 1 week of sponsoring by the number of participants included following that week. We have done this before in two studies to compare the cost of participants from general practice with participants from Facebook, and participants recruited from Facebook cost approximately half that of those from general practice [16, 27].

GPs will invite patients to be included in the trial during their normal consultations with patients with pain under the heel. The general practices will send the contact information of patients to the project manager, whereas potential participants recruited through Facebook will contact the project manager directly. The project manager will then inform the physiotherapists about the potential participant, whom they will contact to perform an eligibility telephone screening. The project manager will have no contact with participants after he has forwarded the contact details to the physiotherapists.

### Eligibility criteria

The inclusion criteria are as follows: history of inferior heel pain for at least 3 months before enrolment, pain on palpation of the medial calcaneal tubercle or the proximal plantar fascia, thickness of the plantar fascia ≥ 4.0 mm, and mean heel pain of ≥ 30 mm on a 100-mm visual analogue scale (VAS) during the previous week. The exclusion criteria are as follows: younger than 18 years of age; diabetes; history of inflammatory systemic diseases (e.g., rheumatoid arthritis or spondyloarthritis) [19]; prior heel surgery; pregnancy or breastfeeding; corticosteroid injection specifically for PF within the previous 6 months; pain or stiffness in the first metatarsophalangeal joint to an extent that the exercises cannot be performed; known hypersensitivity to corticosteroids or local anaesthetics; skin or soft tissue infection near the injection site; receipt of any treatment by a healthcare professional for PF within the previous 12 weeks; or made any substantial changes to usual self-care of the condition in the last 4 weeks (e.g., started using insoles, started performing stretching, made a substantial decrease in physical activity level). These criteria are in line with those of similar studies in this patient population [16, 17, 19]. These criteria lead to a representative sample of patients with PF because previous studies include the majority of potentially eligible participants [16, 17, 19].

### Randomisation and blinding

Participants will be stratified by sex and block-randomised in random concealed block sizes of 3 to 12 (1:1:1) in three parallel groups. A researcher not involved in the trial generated the allocation sequence using a random number generator on www.sealedenvelope.com and is the only person who knows the block sizes. The researcher was trained by the project manager in generating allocation sequences, and the process was piloted.

The randomisation is coded so that the project manager does not know which intervention is linked to which group number (group 1, 2 or 3). The envelopes will be kept in a locked room at Aalborg University Hospital where only the two physiotherapists involved in baseline testing have access. The randomisation schedule was prepared at the Center for General Practice at Aalborg University by a person not involved in the actual trial. The notes in the envelopes state both group number and intervention, and the physiotherapists responsible for assessing participants and delivering the interventions will not be aware of the coding before they open the first envelopes. In practice, after a participant has been enrolled, has filled out questionnaires, and has received initial patient advice and information regarding the practicalities of participation, the physiotherapist will take an envelope and assign the participant to the allocated treatment on the basis of randomisation.

The project manager will be responsible for performing the statistical analyses and will remain blinded to the coding until after the analyses have been performed. The analyses will be performed after the examination that includes the primary endpoint (the 12-week follow-up) of the last participant.

### Interventions

#### Patient advice and heel cup

Participants in all three groups will receive the same initial fundamental patient advice and leaflet about their condition before randomisation. They receive brief information about pathology, risk factors and advice on how to decrease activities that lead to symptom flares and slowly progress back to former activity levels guided by symptoms. To ensure that all participants receive the exact same information, the physiotherapists read a written information out loud; however, they will ask control questions to clarify if the participants understand the information and allow participants to pose questions about the information and participation in general. The leaflet includes the same information that the physiotherapists deliver orally after inclusion in the trial.

Participants will be asked to refrain from seeking other treatments during the course of the trial. They will be allowed to self-treat their PF for pain relief (e.g., with ice or heat packs, rolling a tennis ball under the heel, or massaging the plantar fascia) if they have been doing this for at least 4 weeks prior to inclusion, but they will not be encouraged to do so. Participants will be handed a project diary in which they are asked to record any treatment that they may have received during the course of the trial, including any type of self-treatment and use of analgesic or anti-inflammatory substances. We will inform participants that follow-up on outcomes is critical, regardless of whether they comply with their allocated treatment. Two weeks after inclusion, the physiotherapist who included the participant will contact the participant to ask if they have any questions regarding the condition, the practicalities of participation, or in relation to performing the exercise depending on randomisation. If participants have not achieved a self-evaluated satisfactory result after 12 weeks, they may discuss other evidence-based treatments (e.g., plantar fascia stretching) with the physiotherapist, but they will be encouraged to continue to comply with their allocated treatment.

We will give all participants a silicone heel cup (Medi-Dyne Healthcare Products, Colleyville, TX, USA) for each shoe, and they are advised to use the heel cups whenever they are wearing shoes. If participants already use an insole or another type of foot orthosis, they are allowed to continue wearing this if they prefer this over the heel cups.

#### Heavy-slow resistance training

**Fig. 1:**
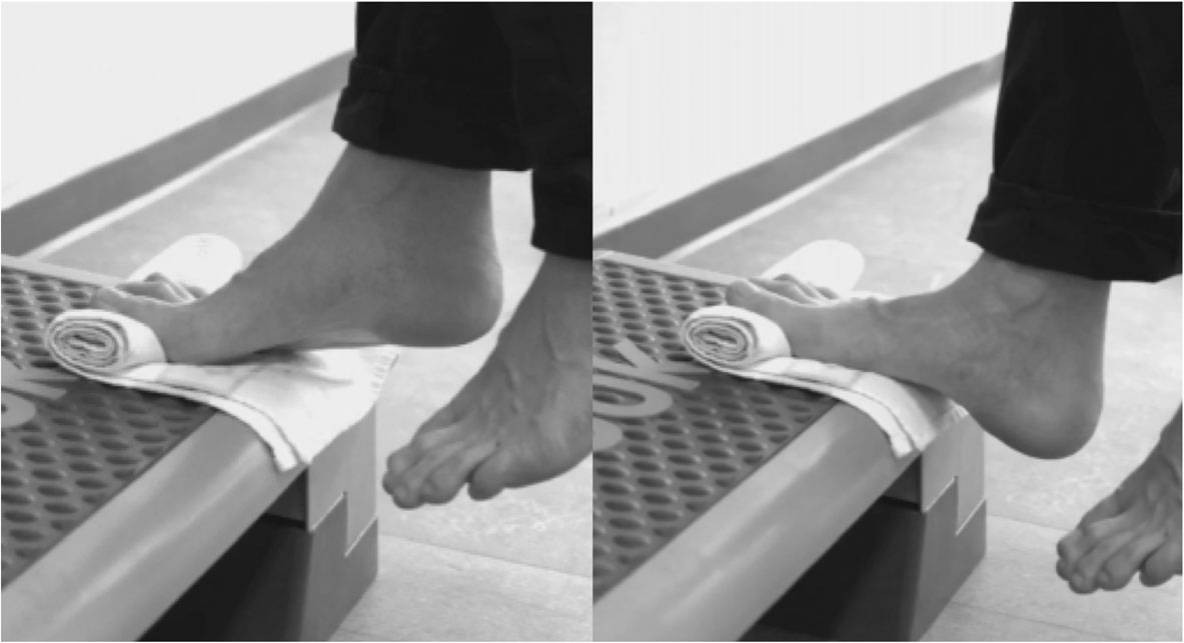
Pictures of the exercise participants receive embedded in the written exercise instruction

Participants of the PAX and PAXI groups will be instructed in performing a heel-raise exercise standing with the forefoot on a step or a book as per Rathleff et al. [26]. Unlike the pre-determined programme used in that trial, we will use a self-dosed programme that we recently found to be associated with the same level of improvement as the pre-determined programme [16]. Participants will be told that it is important that the exercise be performed with an adequately heavy load, and they will be instructed in performing the heel raise with a load corresponding to an 8 repetition maximum (RM) (i.e., a weight that can only be lifted eight times). They may only use a lower relative load if they feel they are unable to perform the exercise with 8 RM. They shall perform the exercise for as many sets as possible. The exercise descriptors are elaborated in Table 1. If participants feel they are able to perform more than eight repetitions with only their body mass (8 RM), an external load consisting of a backpack with books, weights or water bottles to add weight must be used. We will tell participants that pain during the exercise is expected and that there is no upper limit of pain they are allowed to experience, as long as they feel it is tolerable. Participants randomised to also receive the injection will be asked not to perform the exercise within 24 h from the injection and not to progress the method used to achieve 8 RM until 2 weeks after the injection. If heel raise without a backpack is sufficient to achieve 8 RM before the injection, participants should not perform the exercise with a backpack until the third week after the injection, regardless of any pain reduction following the injection. They will be told to perform the exercise until they achieve their Patient Acceptable Symptom State (PASS) (please see ‘Secondary outcomes’ section for elaboration) and an additional 4 weeks. To support the exercise execution, participants receive a written exercise instruction that also includes pictures of the exercise (Fig. 1).

**Table 1:** Exercise descriptors

1. Load magnitude	8 repetition maximum
2. Number of repetitions	≥ 8 depending on the load
3. Number of sets	As many as possible
4. Rest between sets	2 min
5. Number of exercise interventions	Every other day
6. Duration of the experimental period	8 weeks
7. Fractional and temporal distribution of the contraction modes per repetition and duration (in s) of one repetition	3 s concentric 2 s isometric 3 s eccentric
8. Rest between repetitions	No
9. Time under tension	8 s/repetition ≥ 64 s/set ≥ 64 s/training session
10. Volitional muscular failure	Yes
11. Range of motion	Full range of motion
12. Recovery time between exercise sessions	48 h
13. Anatomical definition of the exercise (exercise form)	The participant stands with the forefoot on a step. The toes are maximally dorsally flexed by placing a towel underneath them. The participant performs a heel raise to maximal plantar flexion in the ankle joint and afterwards lowers the heel to maximal dorsal flexion. Supporting oneself for balance by placing the hands on a wall or a rail is allowed.

#### Ultrasound-guided corticosteroid injection

**Fig. 2:**
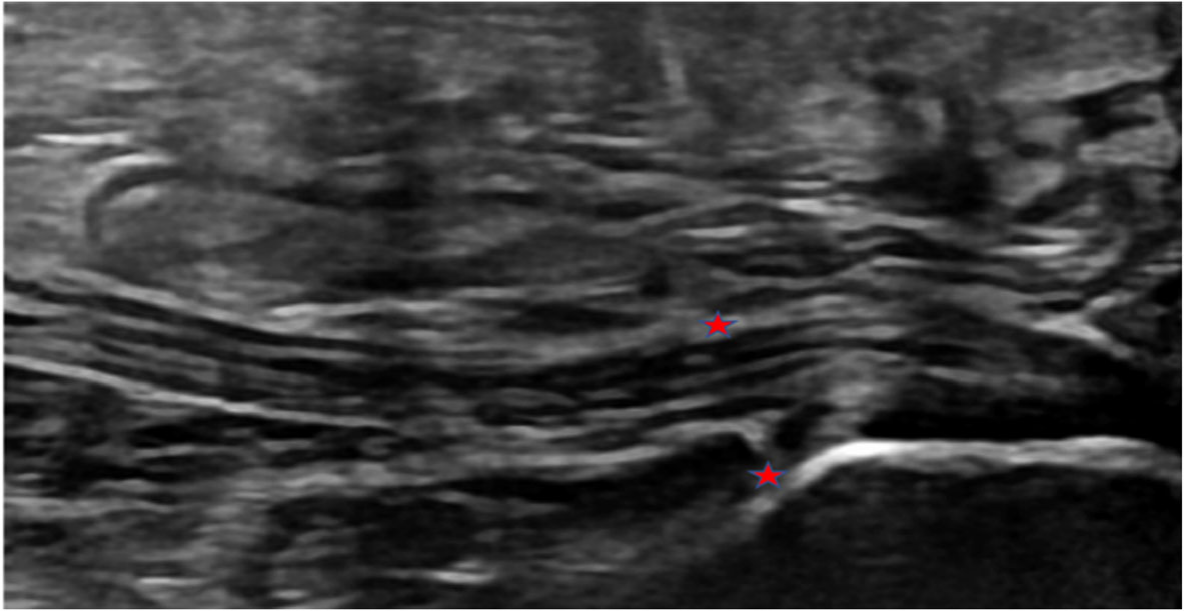
Ultrasound image of the calcaneus and the plantar fascia. The stars depict the placement of the injection

Participants randomised to PAXI will receive an ultrasound-guided corticosteroid injection, preferably within 8 days from baseline but no later than 14 days after baseline. A 21-gauge, 40-mm needle is connected to a 2.5-cm^3^ syringe filled with 1 ml of triamcinolone acetonide 20 mg/ml (Trica; Evolan Pharma, Danderyd, Sweden) + 1 ml of lidocaine 10 mg/ml (Xylocaine; AstraZeneca, Wilmington, DE, USA). The skin is cleansed with chlorhexidine alcohol 0.5% (Medic, Jacksonville, FL, USA). The needle is inserted with a medial approach under ultrasound guidance aligned with the long axis of the ultrasound transducer. The injection is distributed deep and superficially on the plantar fascia surface anterior to the insertion on the calcaneal bone in the region of maximal fascia thickness (see Fig. 2).

### Compliance

We will emphasise to participants that we do not know which of the three groups will be superior and that it is very important to comply with the group to which they are allocated for the future results to be meaningful. Participants in the PAX and PAXI groups will be told that complying with the exercise programme is very important and that exercise compliance is associated with the odds of their recovery. Complying with the exercise programme includes performing the exercise with the prescribed form, contraction time, and sufficient load and frequency. All participants will be asked to record their use of the heel cup and any other foot orthoses in a foot orthoses diary as an estimated percentage of the time they have worn shoes. In addition, participants performing exercise will receive a training diary in which they record the number of repetitions and sets and the date on which the exercise was performed.

### Variables

#### Descriptive

**Fig. 3:**
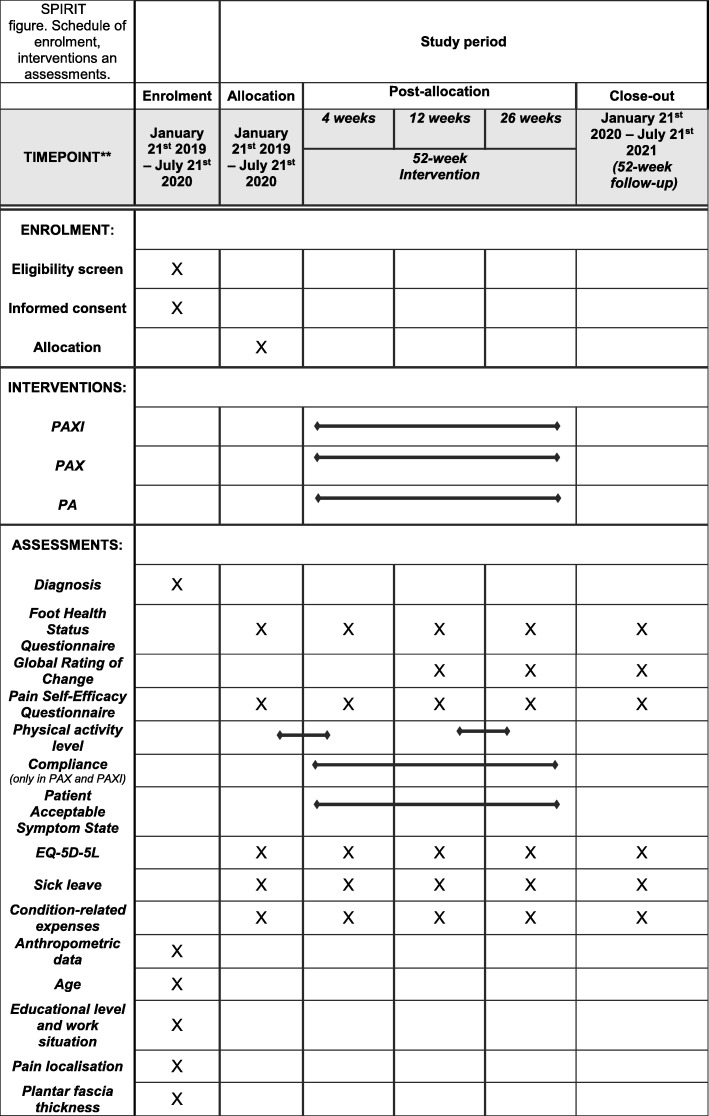
SPIRIT figure. Schedule of enrolment, interventions and assessments

The assessment schedule is found in the SPIRIT figure (Fig. 3). During the telephone screening and the clinical examination, we will collect the following data: age, height, body mass index, location and duration of heel pain, average heel pain intensity during the past week (0 to 100-mm VAS, where 0 is no pain and 100 is worst heel pain imaginable), plantar fascia thickness measured by ultrasonography perpendicular to the calcaneal insertion, presence of palpable pain under the plantar heel, comorbidity, treatment history, previous care-seeking behaviour, if females are pregnant or breastfeeding, number of PF episodes, education level, and work status.

#### Primary outcome

The primary outcome is the mean pain domain score of the FHSQ at the 12-week follow-up. The FHSQ is a questionnaire ranging from 0 (worst possible score) to 100 (best possible score) with high reliability (ICC, 0.74–0.92) that assesses multiple dimensions of foot-related health and function and is recommended in this patient population [35, 36]. The minimal clinically important difference of the pain domain is 14.1 points [37]. We will use a Danish validated translation of the original questionnaire [38].

#### Secondary outcomes

Secondary outcomes include (1) the other domains of the FHSQ (function, footwear and general foot health domains), (2) Global Rating of Change (GROC), (3) PASS, (4) Pain Self-Efficacy Questionnaire (PSEQ), and (5) weekly light, moderate and vigorous physical activity levels.

We will use the GROC to measure participants’ self-reported improvement on a 7-point Likert scale ranging from ‘much improved’ to ‘much worse’. Participants are dichotomised as improved if they rate themselves as ‘much improved’ or ‘improved’ (categories 6 and 7) and categorised as not improved if they rate themselves from ‘slightly improved’ to ‘much worse’ (categories 1 to 5). PASS (yes/no) will be used as a measure of when participants achieve a self-evaluated satisfactory result and feel no need for further treatment. Therefore, this is not necessarily a measure of complete recovery, because some may be satisfied despite still experiencing symptoms. PASS has been used to evaluate clinically relevant states in PF and in other musculoskeletal disorders and post-operative pain [16, 39–41]. Participants will be asked to report to the physiotherapists as soon as they experience PASS, and the date will be noted. Furthermore, participants will be asked about their PASS status during follow-up. After the participant reports a PASS, they will be instructed to continue performing the exercise as prescribed for at least 4 weeks. The PSEQ measures pain self-efficacy and provides a score ranging from 0 (not at all confident) to 60 (completely confident), with lower scores indicating lower self-efficacy [42]. The Danish version of the PSEQ has been validated in a Danish chronic pain population and has high reliability (ICC, 0.89) [43]. To estimate weekly physical activity level expressed as metabolic equivalents (METs), we will use 3D accelerometry. Participants will be given a wrist-worn accelerometer (ActiGraph wGT3X-BT; ActiGraph LLC, Pensacola, FL, USA) during baseline and will be asked to wear this during the first 3 weeks after baseline and then return the accelerometer in a postmarked envelope. During the 12-week follow-up, participants receive the accelerometer again and will be wearing it for an additional 3 weeks before returning it. Participants will be instructed to wear the accelerometer at all times. We will use data from the first valid week recorded during the first and second rounds of wearing the accelerometer (i.e., 1 week during weeks 1 to 3 and 1 week during weeks 13 to 15). A valid week is defined as ≥ 4 days of ≥ 10 h of wear time [44]. Data will be extracted from the accelerometers using the ActiLife software.

#### Cost-effectiveness outcomes

A health economic evaluation will be conducted according to international guidelines [45, 46]. All clinical and cost data will be collected alongside the trial. A health sector perspective will be applied to estimate cost utility using the EuroQol Health Outcome EQ-5D-5L instrument and the Danish quality-adjusted life-year (QALY) weights to calculate gained QALYs within a 1-year horizon [47, 48]. For the estimation of patient-specific costs, we will apply the unique Danish civil registration number for each participant to combine registrations of all healthcare consumption from 1 year before enrolment to 1-year follow-up. Data will be taken from the National Patient Register, the National Health Insurance Register, and the Danish National Prescription Registry. Productivity costs will be estimated in a separate analysis measured by a self-developed questionnaire with questions regarding days of sick leave and level of productivity. Patients’ co-payments and other condition-related expenses will also be estimated by using questionnaires during all follow-ups.

### Adverse events

Participants will be asked to report any adverse events to the physiotherapists immediately after they occur by either telephone, text message or e-mail. Expected adverse events due to the injection are plantar fascia rupture, signs of infection (e.g., fever and local swelling and redness), and local pain in the area of injection lasting more than 48 h after injection. Adverse events after the palpation-guided injection are rare, and two trials that used ultrasound-guided injections reported that no adverse events occurred [19–21]. No stopping rules are planned. Expected adverse events due to the exercise are injuries to the musculoskeletal system, such as muscle tears, muscle strains, a sprained joint, injury from falling or exacerbation of symptoms related to PF, delayed-onset muscle soreness equal to or greater than 20 mm on a 0 to 100-mm VAS that lasts for more than 48 h after performing the exercises, or exacerbation of PF.

Adverse events will be graded 1 to 5 according to the Common Terminology Criteria for Adverse Events v4.03 [49]. A medical doctor specialised in either rheumatology or general medicine will assess and grade the adverse event and ultimately make the decision whether the participant should be withdrawn from the trial due to the adverse event. If the adverse event is a grade 1 (mild), the participant may be allowed to skip one or two training sessions without any assessment. If the adverse event recurs after having skipped the exercise, the participant will have to be assessed by the medical doctor before participation in the trial is continued. If a participant experiences an adverse event and requests withdrawal from the study, data until the last exercise activity before the adverse event occurred will be included in the analyses. The physiotherapists will report any incidents to the sponsor as quickly as possible and no later than 15 days after the participant reported the event. The sponsor will report any severe adverse events (grade 3–5) to the Ethics Committee of North Denmark Region no later than 7 days after being informed. All adverse events will be reported in the future reporting of the trial. Any participants who experience harm from trial participation will receive compensation by the Patient Compensation Association.

### Concurrent observational cohort

Potential participants who are excluded during the physical examination and eligible participants who decide to withdraw before randomisation will be asked to be part of a concurrent observational cohort inspired by the ﻿Spine Patient Outcomes Research Trial [50]. This cohort will be used to describe how excluded participants fare and which treatment they seek. If they agree to be in the cohort, they will receive the same questionnaires as the participants of the FIX-Heel Trial with the addition of a questionnaire about care-seeking behaviour and treatments received during the time between the last follow-up and the current follow-up. We will use the same follow-up times (4, 12, 26 and 52 weeks) as in the FIX-Heel Trial; however, all follow-ups will be conducted through e-mail.

### Patient and end-user involvement

To involve both patients and end users in designing the intervention, the participant leaflet was developed on the basis of semi-structured interviews with five patients with PF and five GPs. Patients were asked to describe their heel pain, how it had affected them and which topics they felt would be important to include in a leaflet and in advice to a patient in general. GPs were asked about their experience with the patient group, which treatments they would consider and which topics they felt would be important to include in a leaflet and in advice to the patient in general. The leaflet and fundamental patient advice that the physiotherapists will deliver orally to patients are a triangulation of the results of the interviews with both patients and GPs and recommendations from clinical guidelines and a systematic review [1, 6, 51].

We will invite representative participants from each group to be part of the future interpretation of the results. Their interpretation will be part of the dissemination of the results in the trial report and during conferences. In practice, we will invite two randomly selected participants of each group after the primary outcome has been collected. If a participant declines, we will randomly select a new participant from the same group.

### Sample size

The minimal clinically important difference of the FHSQ pain domain has been found to be either 12.5 or 14.1 points in this patient population [37, 52]. We have chosen the most conservative option (i.e., 14.1 points) to form the basis of the sample size calculation. Based on a standard deviation of 22 points, which is comparable to the overall standard deviations found in previous studies of this patient population [19, 53, 54], a two-sided 5% significance level, and a power of 90%, a sample size of 53 participants in each group will be necessary. Taking into consideration possible drop-outs, we will include 60 participants in each group, and thus the total sample size will be 180 participants.

### Statistical analyses

All statistical analyses will be performed by a blinded data analyst according to a pre-established analysis plan using the intention-to-treat principle. This plan is written in consultation with a statistician and will be published on the Aalborg University website before the inclusion of the last participant. SPSS software (IBM Corporation, Armonk, NY, USA) will be used as statistical software. We will use Q-Q plots and histograms to assess data normality. Missing outcome data will be imputed using multiple imputations based on the values from previous follow-ups, sex, age, and group allocation.

#### Primary analysis

The primary analysis will investigate the between-group difference in FHSQ pain. We will use a linear mixed effects model with the participant as a random effect, and time (4, 12, 26 and 52 weeks), group allocation (PA or PAX or PAXI) and baseline value as fixed effects. Conclusions will only be drawn on the basis of the primary endpoint (12 weeks).

#### Secondary analyses

We will also analyse the mean values of the secondary continuous outcomes (other domains of FHSQ, PSEQ and physical activity level) using linear mixed models. The risk difference ($=\frac{Positive\ outcomes\ in\  one\  group}{Number\ of\ participants\ in\ group}$ - $\frac{Positive\ outcomes\ in\ another\ group}{Number\ of\ participants\ in\ group}$) will be calculated for the dichotomised GROC to determine the probability of being improved, for the PASS (yes/no) to determine the probability of achieving a self-evaluated satisfactory result within the 12, 26 and 52 weeks of intervention. We will also calculate risk differences to determine the probability of experiencing a deterioration of PF, defined as a decrease in FHSQ pain ≥ 14.1 points from one follow-up to another or changing one’s status from having achieved PASS to no longer having achieved PASS. We will calculate the number needed to treat for the primary outcome at the primary endpoint as 1/risk difference. We will use a Kaplan-Meier survival analysis and compare survival curves using log-rank tests to investigate between-group differences in time to achieving PASS [55, 56]. If a participant changes PASS multiple times (e.g., achieving PASS before 12 weeks, reporting not to have achieved PASS at 26 weeks and then having achieved PASS again at the 52-week follow-up), only time to the first PASS achieved is used in the analysis.

#### Cost-effectiveness analysis

The reporting of the economic evaluation will follow the Consolidated Health Economic Evaluation Reporting Standards checklist for a more transparent and complete reporting of methods and findings [57]. For each intervention, mean values (and standard errors of the mean) will be reported for the main categories of estimated costs and QALYs, as well as mean differences between the comparator groups. Probabilistic sensitivity analyses will be used to estimate the decision uncertainty and calculate incremental cost-effectiveness ratios.

### Data monitoring and quality assurance

All data will be stored electronically and are handled according to the General Data Protection Regulation. Data safety may be overseen unannounced by the Danish Data Protection Agency. Participant data will be stored in REDCap, whereas data processor agreements, collaboration agreements between the project group and general practices and protocols will be stored on a secure server at Aalborg University. To prevent data entry errors, data collection instruments have been developed in REDCap so that required data must be included or an error will be displayed, and validation of each field has been chosen (e.g., if the format of the data does not appear to be a date in the field ‘Date’, an error is displayed). Data are checked once per week by the project manager to ensure there are no missing data. All data will be kept for 10 years after completion of the trial in accordance with the European Code of Conduct for Research Integrity.

## Discussion

### Future implications

PF is a condition with a wide variety of different treatment options available, with no single treatment showing superiority [18, 51]. HSR is increasingly being used for rehabilitation of PF, despite its effects having only been compared with stretching in a single study [26]. Our recent research with HSR for PF shows within-group improvements in pain similar to those of studies investigating foot orthoses, corticosteroid injections and even placebo injections [16]. By comparing HSR with no HSR and a heel cup and patient advice only, we will be able to answer if performing exercises is worth the extra time and effort required from the patient.

Corticosteroid injection may reduce heel pain for up to 1 month, and adverse events are generally rare and have been reported not to occur when the injection was performed under ultrasound guidance [19–21]. After 1 month, the improvement in symptoms is similar to that of placebo. In contrast, the effects of HSR are known for taking some time to manifest, and performing HSR is usually painful [16, 17, 26]. Patients ask for both short-term and long-term pain reduction, and the combination of these two treatments may potentially offer this. Moreover, in the qualitative data from our feasibility study, we found that several participants felt that pain during exercise was reduced due to the pain relief associated with the injection [27]. This may improve exercise compliance and overall improvement. Despite our hypothesis of superiority of combining the injection with HSR, an injection with corticosteroid may potentially reduce the effect of HSR. This would be similar to how corticosteroids reduced the effect of physiotherapy in a former trial in patients with lateral elbow tendinopathy. In that trial, corticosteroid injections and physiotherapy led to a lower rate of successful outcomes than placebo injections and physiotherapy after 1 year [24]. This indicates that corticosteroids decreased the effect of physiotherapy. Furthermore, the combination of exercise and an injection was not superior to an injection alone in patients with subacromial pain syndrome [29]. One concern is that the pain reduction following the injection will hamper exercise compliance and affect long-term outcomes negatively.

The implementation of our findings will be aided by the inclusion of a cost-effectiveness analysis. There are obvious differences between treatments in how much time and materials are required. The treatment offered in the PA group requires the least, whereas the PAXI group requires the most. However, this difference may be equalised by the potential savings on a societal level in terms of sick leave or on a personal level in terms of QALYs, condition-related improvement and personal expenses. Any future implementation will also be dependent on the patients’ experiences and expectations, which is why patients are involved in the interpretation of the trial findings.

Recruiting patients for trials in primary care is a large challenge, and less than one-third of trials recruit patients within the original recruitment time frame [58]. We use an adaptive recruitment strategy in which we focus primarily on recruitment from general practices, but we will use Facebook recruitment to ensure that the timeline is kept. We may increase the number of recruiting practices or the area and the money used for sponsoring Facebook posts, depending on recruitment rate. This may help inform effective ways of recruiting a large number of participants from general practice in studies requiring large numbers of patients.

### Strengths

First, the involvement of patients and GPs in developing the patient advice intervention enables a high level of acceptability for users. Second, all of the interventions are within scope, knowledge and skill levels of primary care clinicians and practices, so they will have a high feasibility of being implemented, regardless of trial findings. Third, all potential outcomes of the trial may influence future clinical practice. If no superiority of one intervention is found over the others, the minimally invasive or the most cost-effective should be implemented, depending on patient preferences. Fourth, to minimise bias, the data analysis will occur blind to group allocation and will be performed according to a pre-established and publicly accessible analysis plan.

### Limitations

First, despite the trial being rooted in general practice, the treatments are delivered at the hospital and at a private rheumatology clinic for logistical reasons by physiotherapists and a rheumatologist. Second, if the PAXI group is superior to the others, we cannot disregard that some of that superiority will derive from the placebo effect of receiving an injection. However, we evaluated how the relevance of adding a placebo injection to the PAX group had lessened in light of research concluding the superiority of corticosteroid injections compared with placebo [19–21]. Third, we recruit from both general practice and from Facebook, and we do not know if these recruitment sources yield the same type of patient; still, based on our previous study where we recruited patients solely via Facebook, three in four will have seen their GP due to their PF [17]. This may increase the generalisability of the patients recruited from Facebook to those seen in general practice.

### Trial status

Recruitment was started on January 21, 2019, and the first participant was included on February 7, 2019. No amendments have been made to the protocol (version 2.0 January 14, 2019) since it was pre-registered with ClinicalTrials.gov. When this protocol was submitted for publication (October 5, 2019), a total of 96 participants had been included in the trial. We expect recruitment to be completed in July 2020.

## Supplementary information

**Additional file 1.** SPIRIT 2013 checklist: recommended items to address in a clinical trial protocol and related documents.

## Data Availability

Data will be made available upon reasonable request.

## Abbreviations

FHSQ: Foot Health Status Questionnaire
GP: General practitioner
GROC: Global Rating of Change
HSR: Heavy-slow resistance training
MET: Metabolic equivalent
PA: Patient advice and heel cup
PASS: Patient Acceptable Symptom State
PAX: Patient advice, heel cup and heavy-slow resistance training
PAXI: Patient advice, heel cup, heavy-slow resistance training and corticosteroid injection
PF: Plantar fasciopathy
PSEQ: Pain Self-Efficacy Questionnaire
QALY: Quality-adjusted life-year
RM: Repetition maximum
SPIRIT: Standard Protocol Items: Recommendations for Interventional Trials
TiDieR: Template for Intervention Description and Replication checklist and guide
VAS: Visual analogue scale
